# N6‐Methyladenosine Modification of circIST1 Promotes Hypoxia‐Inducible Factor α–mediated Glycolysis and Progression in Hepatocellular Carcinoma

**DOI:** 10.1002/mco2.70577

**Published:** 2026-01-14

**Authors:** Yangyang Zhan, Zhongmin Wang, Fei Teng, Qian Ding, Lei Lv, Fangyuan Xie, Yueying Huang, Xue Jiang, Dan Zheng, Xiaoying Ge, Shuqun Cheng, Yizhun Zhu, Leilei Bao

**Affiliations:** ^1^ Department of Pharmacy Shanghai Eastern Hepatobiliary Surgery Hospital, Navy Military Medical University Shanghai China; ^2^ Department of Oncology Shanghai East Hospital, Tongji University School of Medicine Shanghai China; ^3^ School of Materials and Chemistry & Institute of Bismuth, University of Shanghai for Science and Technology Shanghai China; ^4^ Department of Liver Surgery and Organ Transplantation Changzheng Hospital, Naval Medical University Shanghai China; ^5^ State Key Laboratory of Quality Research in Chinese Medicine School of Pharmacy, Macau University of Science and Technology Macau China; ^6^ Department of Hepatic Surgery VI Eastern Hepatobiliary Surgery Hospital, Navy Military Medical University Shanghai China

**Keywords:** circIST1, hepatocellular carcinoma, HIF‐1α, m6A modification, microRNA sponge, tumor glycolysis

## Abstract

The involvement of circular RNAs (circRNAs) have been well‐documented in various cancers, including hepatocellular carcinoma (HCC); however, their regulatory roles in HIF‐1α‐mediated tumorigenesis remain largely unclear. This study elucidates the functional significance of N6‐methyladenosine (m6A)‐modified circRNA—circIST1 in HCC progression. Elevated expression of circIST1 was observed in both HCC clinical specimens and cultured cell lines. This pronounced upregulation was found to be associated with poor prognosis and survival. Functionally, circIST1 drives HCC progression by enhancing tumor cell proliferation, migration, and invasion and by inhibiting apoptosis, as validated in vitro and in vivo. Mechanistically, it functions as a competitive endogenous RNA (ceRNA) that sponges miR‐140‐3p and miR‐182, thereby relieving their repression on the common downstream oncogene, HIF‐1α. Rescue experiments confirm that the tumor‐suppressive effects of circIST1 silencing are reversed upon inhibition of these miRNAs or overexpression of HIF‐1α. Notably, we show that circIST1 drives HIF‐1α‐mediated aerobic glycolysis—a metabolic hallmark of cancer—‐by enhancing glucose uptake, lactate production, and glycolytic flux. Furthermore, we identify methyltransferase‐like 3 (METTL3)‐dependent m6A modification as a critical regulator of circIST1 stability. Collectively, our findings uncover a novel m6A‐circIST1‐miR‐140‐3p/miR‐182‐HIF‐1α regulatory axis that underlies metabolic reprogramming in HCC, positioning circIST1 as a promising therapeutic target for HCC metabolic intervention.

## Graphical abstract

We identified first time a critical m6A modification of circIST1 on miR‐140‐3p/miR‐182‐HIF1α regulatory network involved in the HCC progression and tumor glycolysis, gaining mechanistic insights into the function of circRNAs in HCC progression.

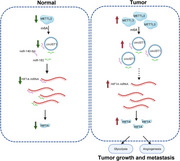

## 1 Introduction

Liver cancer poses a major global health burden; recent statistics indicate it is the sixth most frequently diagnosed cancer and the third leading cause of cancer‐related mortality worldwide [1]. Hepatocellular carcinoma (HCC), which accounts for approximately 85% of primary liver cancer cases, is characterized by an increasing incidence and high mortality rate, driven by late diagnosis, intrinsic tumor heterogeneity, and the rapid development of therapeutic resistance [2, 3]. Although the introduction of targeted agents and immune checkpoint inhibitors—such as atezolizumab (anti‐PD‐1) in combination with bevacizumab (anti‐VEGF)—has significantly improved survival outcomes in advanced HCC, overall clinical responses remain suboptimal, with an objective response rate of only 27.3% [4]. Moreover, HCC exhibits considerable heterogeneity and rapidly progresses with the emergence of drug resistance, often resulting in a lack of effective treatment strategies [5]. This clinical urgency underscores the need to identify novel molecular regulators that drive HCC pathogenesis, particularly those involved in early tumor progression and metabolic adaptation. Thus, the exploration of novel therapeutic targets remains a critical challenge in HCC research. This study explored circIST1, a novel circRNA with uncharacterized function and mechanism in HCC, suggesting it is a promising therapeutic target.

CircRNAs are a class of long noncoding RNAs characterized by a covalently closed circular structure lacking 5′ and 3′ ends [6]. Accumulating evidence indicates that circRNAs exhibit tissue‐specific and tumor‐specific expression patterns and play critical roles in regulating malignant proliferation, metastasis, metabolism, immune evasion, and other tumor‐related processes, thereby demonstrating increasing potential in tumor diagnosis and therapy [7]. The predoinvolveolecular mechanism of circRNAs involves acting as microRNA (miRNA) sponges, where they sequester miRNAs and thereby relieve the inhibitory effects of miRNAs on their downstream targets [8, 9]. Although comprehensive studies have revealed diverse functional roles of circRNAs, they are still primarily recognized as regulators of gene expression. In this study, by analyzing three distinct circRNA expression profiles in HCC, we identified a novel HCC‐associated circRNA, named circIST1, and demonstrated that circIST1 modulates the expression of the key oncogene HIF‐1α through regulating two different miRNAs. Hypoxia‐inducible factor 1 (HIF‐1) is a heterodimeric transcription factor consisting of HIF‐1α and HIF‐1β subunits. It governs the expression of a broad spectrum of genes implicated in key cancer hallmarks, including angiogenesis, cell survival, metabolic reprogramming, and invasion [10, 11]. Consequently, interest in identifying novel regulators and small‐molecule inhibitors of HIF‐1 has grown exponentially in recent years. HIF‐1α, as a critical subunit of HIF‐1, is essential for its transcriptional activity [12]. The regulation of HIF‐1α expression is currently classified into two categories: one is oxygen‐dependent, primarily mediated by FIH‐1 (factor inhibiting HIF‐1); the other is oxygen‐independent, regulated by signaling pathways such as PI3K/AKT/mTOR [13]. However, accumulating evidence indicates that post‐transcriptional regulation, particularly by non‐coding RNAs, plays an increasingly recognized role in stabilizing HIF‐1α under normoxic conditions—a phenomenon known as “pseudohypoxia” that is commonly observed in HCC [14]. Nevertheless, the oxygen‐independent regulatory mechanisms of HIF‐1α remain to be fully elucidated. In our study, we demonstrate that circIST1 functions as a novel oxygen‐independent regulator of HIF‐1α, specifically acting as a positive post‐transcriptional regulator.

Tumor cells exhibit a distinct metabolic preference for converting glucose into lactate even under oxygenated conditions, a phenomenon termed aerobic glycolysis or the “Warburg effect” [15]. This metabolic reprogramming is crucial for maintaining malignant growth and metastasis, making its inhibition a promising anticancer strategy [16]. This metabolic shift not only supports rapid ATP production and the accumulation of biosynthetic precursors but also contributes to an immunosuppressive tumor microenvironment (TME), thereby facilitating immune evasion [17]. Previous studies have shown that targeting circQSOX1 to suppress aerobic glycolysis in colorectal cancer can reduce the expansion and infiltration of regulatory T cells (Tregs), thus enhancing anti‐tumor immune responses [18]. These findings underscore the therapeutic potential of targeting circRNA‐mediated metabolic reprogramming. In this study, we report a novel mechanism for attenuating HIF‐1α‐mediated aerobic glycolysis by targeting circIST1, suggesting a new avenue for metabolic therapy in HCC.

Accumulating evidence indicates that the m6A modification critically influences multiple aspects of circular RNAs (circRNAs) in cancer, including their biogenesis, stability, and functional activity [19]. As the most abundant internal mRNA modification, m6A is dynamically regulated by “writers” (e.g., METTL3), “erasers” (e.g., FTO, ALKBH5), and “readers” (e.g., YTHDFs, IGF2BPs), and has been shown to enhance the stability and oncogenic functions of certain circRNAs [20]. In HCC, dysregulation of m6A modifiers is frequently observed and is associated with poor prognosis [21]. However, whether m6A modification regulates circIST1 and contributes to its oncogenic activity remains unknown. Our study reveals that circIST1 undergoes extensive METTL3‐dependent m6A modification. This epigenetic mark enhances circIST1's RNA stability and functional activity. Consequently, these findings add a crucial regulatory layer to the circIST1‐miR‐140‐3p/miR‐182‐HIF‐1α axis, offering deeper mechanistic insight into HCC.

## 2 Results

### 2.1 circIST1 is Upregulated in HCC and Correlated With Poor Prognosis

**FIGURE 1:**
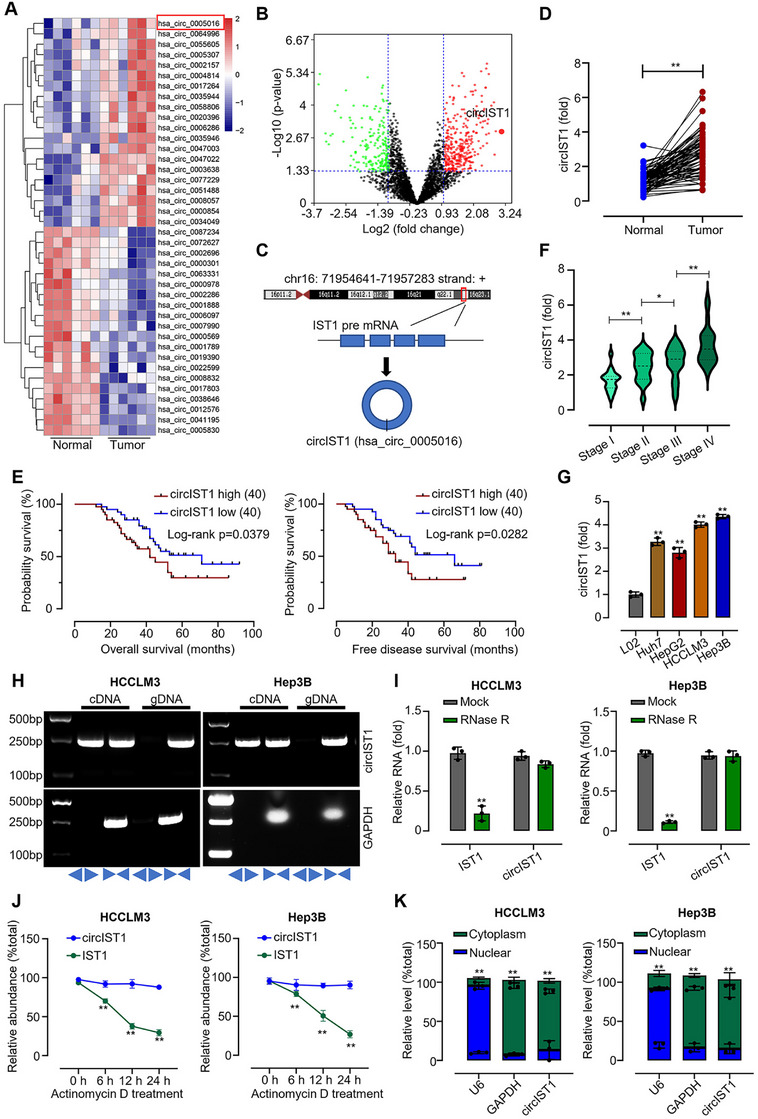
circIST1 is upregulated in HCC tissues and cells. (A) Heatmap of differentially expressed circular RNAs in six HCC patients and six normal volunteers according to the online data set (GSE164803, absolute log2 fold change > 1, *p* < 0.05); (B) a volcano map of differential genes; (C) chromatin location of circIST1; (D) fold change of circIST1 levels in cancer samples compared to para‐cancer samples; (E) Kaplan–Meier analysis was used to analyze the association between expression of circIST1 and the overall/free disease survival rate of HCC patients; (F) fold change of circIST1 levels in patient samples with different stages; (G) fold change of circIST1 levels in one human normal hepatic epithelial cell line L02 and various HCC cell lines, Huh7, HepG2, HCCLM3, and Hep3B; (H) DNA levels of circIST1 amplified by the specific primers from functional regions of circIST1 or common primers in HCCLM3 and Hep3B cells; (I) expression levels of circIST1 in HCCLM3 and Hep3B cells treated with Rnase R; (J) relative abundance of circIST1 in HCCLM3 and Hep3B cells induced by actinomycin D (5 µM) in 24 h; (K) mRNA levels of U6 (nuclear control), GAPDH (cytoplasmic control), and circIST1 were measured using qRT‐PCR in HCCLM3 and Hep3B cells. Data are representative of three independent experiments and shown as mean ± SD. **p < 0.01.

To explore circRNA function in HCC, differentially expressed circRNAs were first identified from the GSE164803 dataset through analysis of six paired HCC and matched adjacent normal tissues (Figure 1A,B). circIST1 (hsa_circ_0005016) was one of the most significantly upregulated circRNAs and has not been previously reported (Figure 1B). circIST1 is located at chr16:71954641‐71957283 and is formed by back‐splicing of exons from its parental gene (Figure 1C). circIST1 expression was further evaluated in clinical samples. CircIST1 was significantly dysregulated in HCC, showing marked upregulation in 80 paired tumor tissues versus adjacent non‐tumor tissues (Figure 1D). Elevated circIST1 levels carried negative prognostic implications, correlating with reduced overall and disease‐free survival, as well as advanced tumor stage (Figure 1E,F; Table S1). Consistent with this, circIST1 expression was higher in multiple HCC cell lines (Huh7, HepG2, HCCLM3, Hep3B) than in the normal hepatic L02 cell line (Figure 1G). Molecular validation using junction‐spanning divergent primers confirmed the circular structure of circIST1 in HCCLM3 and Hep3B cells, whereas linear GAPDH was amplified only by convergent primers (Figure 1H). To confirm the circular structure of circIST1, HCCLM3 and Hep3B cells were treated with ribonuclease R (RNase R). Subsequent qRT‐PCR analysis showed that RNase R robustly degraded linear IST1 mRNA but did not significantly reduce circIST1 levels (Figure 1I). Additionally, HCCLM3 and Hep3B cells were treated with actinomycin D for 6, 12, and 24 h to assess transcript stability. qRT‐PCR analysis revealed that circIST1 exhibited a longer half‐life than its linear counterpart IST1 (Figure 1J). A well‐established role of circRNAs is to serve as ceRNAs, which sequester miRNAs and impair their function [22, 23, 24, 25, 26]. Subcellular fractionation followed by qRT‐PCR indicated that circIST1 is predominantly localized in the cytoplasm of HCCLM3 and Hep3B cells (Figure 1K), suggesting a potential role for circIST1 as a ceRNA.

### 2.2 m6A Serves as a Critical Determinant of circIST1's Oncogenic Activity in HCC

**FIGURE 2:**
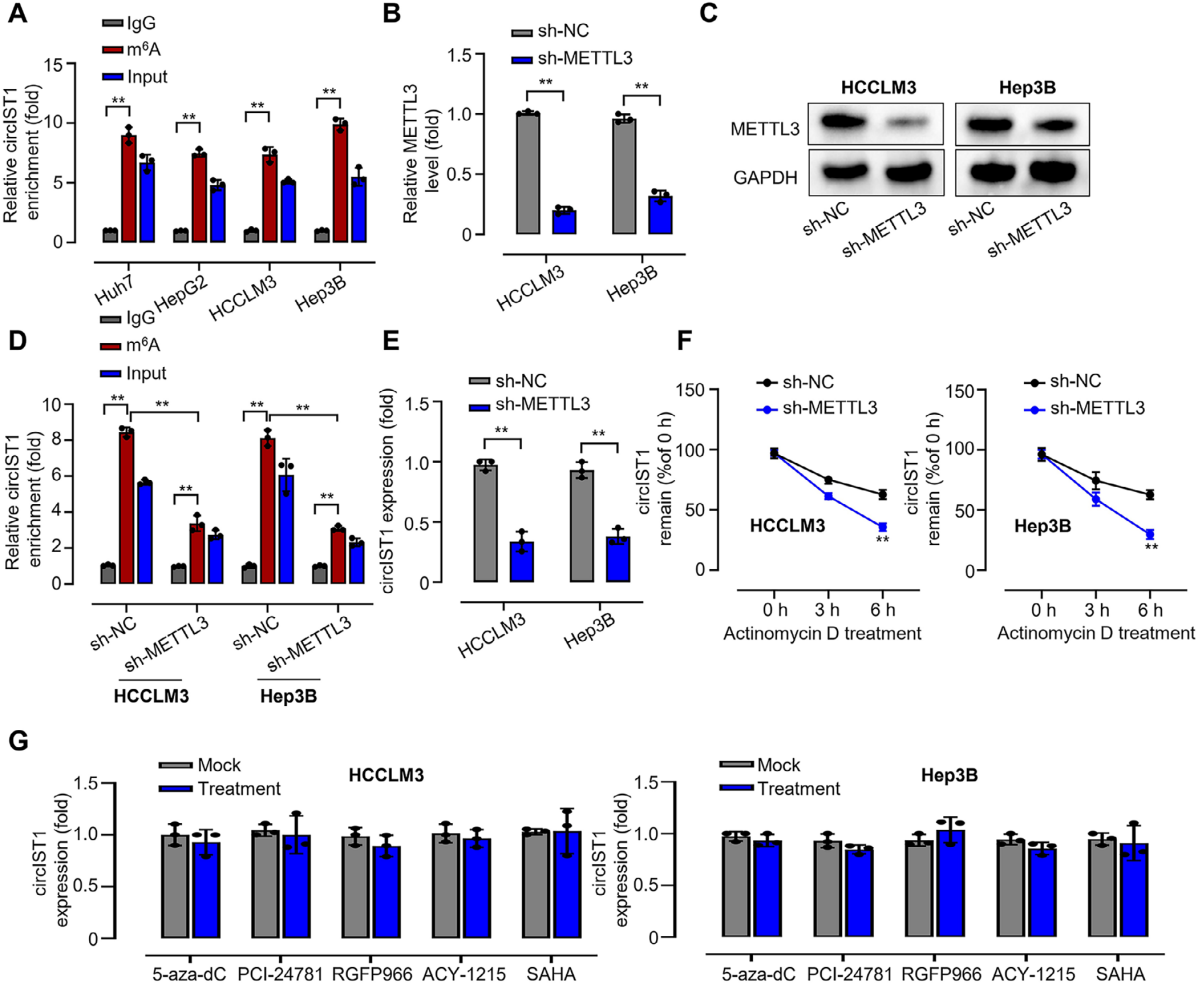
m6A is essential for the function of circIST1 in HCC. (A) Relative circIST1 enrichment on m6A in Huh7, HepG2, HCCLM3, and Hep3B cells; (B) mRNA and (C) protein levels of METTL3 in HCCLM3 and Hep3B cells transfected with sh‐NC or sh‐METTL3; (D) relative circIST1 enrichment on m6A in HCCLM3 and Hep3B cells transfected with sh‐NC or sh‐METTL3; (E) relative mRNA expression of circIST1 in HCCLM3 and Hep3B cells transfected with sh‐NC or sh‐METTL3; (F) relative remaining circIST1 in HCCLM3 and Hep3B cells induced by actinomycin D in 6 h; (G) relative mRNA expression of circIST1 in HCCLM3 and Hep3B cells treated with 5‐aza‐dC, PCI‐24781, RGFP965, ACY‐1215, and SAHA. Data are representative of three independent experiments and shown as mean ± SD. **p < 0.01.

m6A methylation is a prevalent post‐transcriptional RNA modification that plays a critical role in regulating physiological processes, yet its functions in circRNA biology remain incompletely understood [27]. We next investigated whether circIST1 is subject to m6A modification. The data showed that m6A is enriched in circIST1 across multiple HCC cell lines (Figure 2A). METTL3 functions as a key m6A methyltransferase in cancer [28, 29, 30]. We knocked down METTL3 in HCCLM3 and Hep3B cells (Figure 2B,C) and found that both m6A modification levels on circIST1 and circIST1 abundance were significantly reduced in METTM3‐depleted cells (Figure 2D,E). Furthermore, actinomycin D chase experiments were performed at 3 and 6 h to assess circIST1 stability. qRT‐PCR analysis revealed that circIST1 exhibited a shorter half‐life in METTL3‐depleted cells compared to controls (Figure 2F); however, due to experimental batch effects, some variability in degradation kinetics was observed. To determine whether DNA or histone deacetylation pathways regulate circIST1, we treated HCCLM3 and Hep3B cells with epigenetic modulators, including 5‐aza‐dC, PCI‐24781, RGFP965, ACY‐1215, and SAHA. Assessment of circIST1 expression by qRT‐PCR following RNA extraction revealed no significant change after treatment (Figure 2G). These data suggest that the upregulation of circIST1 in HCC is mediated by METTL3‐dependent m6A methylation rather than by transcriptional or chromatin‐level regulation.

### 2.3 circIST1 Promotes Proliferation, Metastasis, and Inhibits Apoptosis of HCC

**FIGURE 3:**
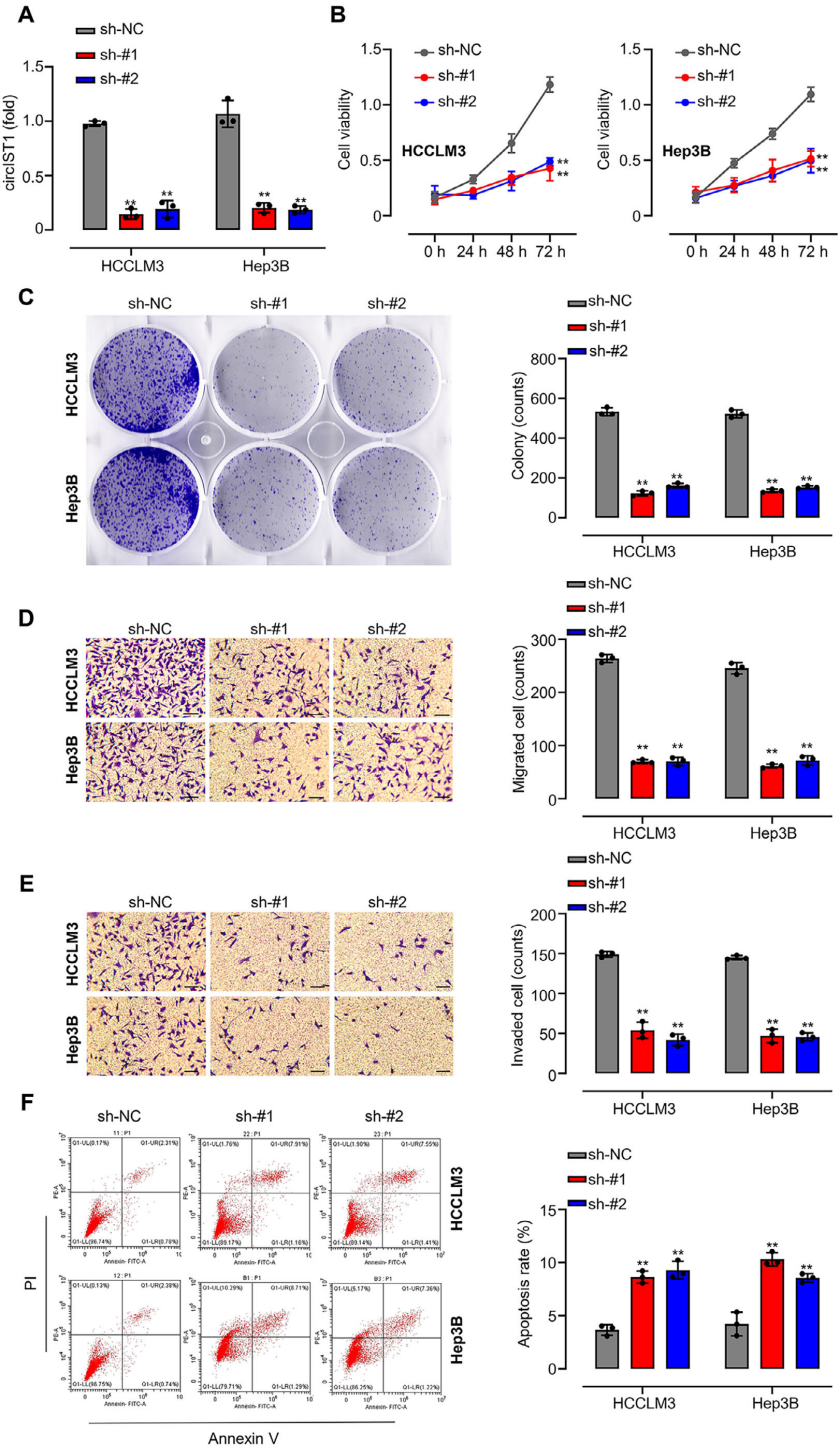
circIST1 downregulation inhibits HCC cell proliferation, migration, invasion, and induces cell apoptosis. (A) qRT‐PCR analysis of circIST1 mRNA levels in HCCLM3 and Hep3B cells transfected with sh‐NC or sh‐circIST1#1 or sh‐circIST1#2; (B and C) CCK8 and colony formation assays were performed to assess the proliferation of HCCLM3 and Hep3B cells transfected with sh‐NC or sh‐circIST1#1 or sh‐circIST1#2; (D and E) Transwell assays were used to assess the migration and invasion abilities of HCCLM3 and Hep3B cells transfected with sh‐NC or sh‐circIST1#1 or sh‐circIST1#2; (F) annexin V/PI staining was used to assess the cell apoptosis of HCCLM3 and Hep3B cells transfected with sh‐NC or sh‐circIST1#1 or sh‐circIST1#2; data are representative of three independent experiments and shown as mean ± SD. **p < 0.01.

To functionally characterize circIST1 in HCC progression, we performed loss‐of‐function studies in HCCLM3 and Hep3B cells, which express high endogenous circIST1 levels (Figure 3A). Its knockdown markedly attenuated cellular proliferation, as evidenced by CCK‐8 assays (Figure 3B) and colony formation assays (Figure 3C). Furthermore, circIST1 depletion concomitantly impaired migratory and invasive capacities in Transwell assays (Figure 3D,E) and increased apoptosis rates as measured by flow cytometry (Figure 3F). The pro‐tumorigenic role of circIST1 was corroborated in vivo; xenograft tumors derived from circIST1‐silenced Hep3B cells exhibited significantly reduced growth in volume and weight compared to controls (Figure S1C). Collectively, these data establish that circIST1 drives HCC progression by enhancing proliferation, migration, and invasion while suppressing apoptosis.

### 2.4 circIST1 Promotes HCC Progression via Sponging miR‐140‐3p/miR‐182

**FIGURE 4:**
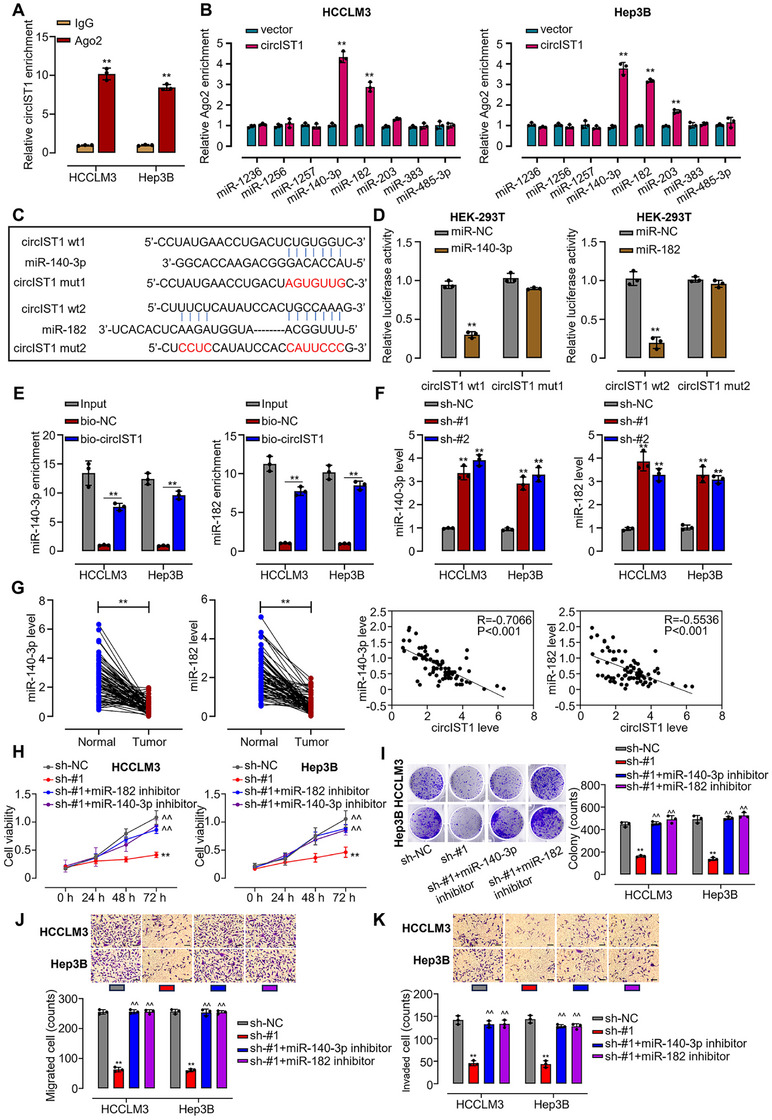
circIST1 promotes HCC cell proliferation, migration, and invasion via sponging miR‐140‐3p/miR‐182. (A) RIP and qRT‐PCR assays were performed to determine the interaction between Ago2 and circIST1 in HCCLM3 and Hep3B cells; (B) RIP‐qRT‐PCR assays were performed to determine the interaction between potential targets of circIST1 and Ago2 in HCCLM3 and Hep3B cells; (C) A predicted binding site of miR‐140‐3p/miR‐182 within circIST1 by bioinformatic analysis using the StarBase 3.0 (http://starbase.sysu.edu.cn/); (D) luciferase reporter assays were used to determine the binding sites between miR‐140‐3p and miR‐182 with circIST1; (E) RNA pull‐down‐qRT‐PCR assay was conducted to determine the interaction between circIST1 and miR‐140‐3p/miR‐182; (F) qRT‐PCR assay was used to detection the miR‐140‐3p and miR‐180 expression in circIST1 knockdown and control HCCLM3 and Hep3B cells; (G) qRT‐PCR assay was used to detect the expression in cancer samples compared to para‐cancer samples (left panel); Pearson correlation analysis was used to analyze the expression correlation between miR‐140‐3p/miR‐182 and circIST1; CCK8 (H) and colony formation (I) assays were utilized to assess the proliferation of circIST1 knockdown HCCLM3 and Hep3B cells, which were simultaneously transfected with miR‐140‐3p/miR‐182 inhibitors; (J–K) we used Transwell assays to assess the migration and invasion of circIST1 knockdown HCCLM3 and Hep3B cells transfected with miR‐140‐3p/miR‐182 inhibitors. Data are representative of three independent experiments and shown as mean ± SD. **p < 0.01, compared to the sh‐NC group; ^^*p* < 0.01, compared to the sh‐circIST1#1 group. sh‐#1 means shRNA circIST1 #1.

Building on the established ceRNA function of circRNAs, we postulated that circIST1 might operate as a miRNA sponge [22, 23, 24, 25, 26]. This was initially supported by its specific interaction with the Ago2 protein (Figure 4A). To identify candidate miRNAs, bioinformatic screening (StarBase) was followed by RNA pull‐down assays in circIST1‐overexpressing cells, which identified miR‐140‐3p and miR‐182 as the most enriched miRNAs bound to Ago2 (Figure 4B). Predicted binding motifs (Figure 4C) and luciferase reporter assays with mutant constructs confirmed direct, specific interactions (Figure 4D, Figure S4). Further corroborating this, a biotinylated circIST1 probe pulled down both miRNAs (Figure 4E), and circIST1 knockdown reciprocally increased their levels (Figure 4F). Clinically, both miRNAs were downregulated in HCC tissues and showed a significant inverse correlation with circIST1 expression (Figure 4G). Crucially, the growth‐suppressive and motility‐impairing effects of circIST1 knockdown were substantially reversed by inhibiting miR‐140‐3p/miR‐182 (Figure 4H–K, Figure S1A). Collectively, these data demonstrate that circIST1 exerts its oncogenic effects in HCC by sequestering miR‐140‐3p and miR‐182.

### 2.5 HIF‐1α Is a Direct Target Gene of miR‐140‐3p and miR‐182 in HCC

**FIGURE 5:**
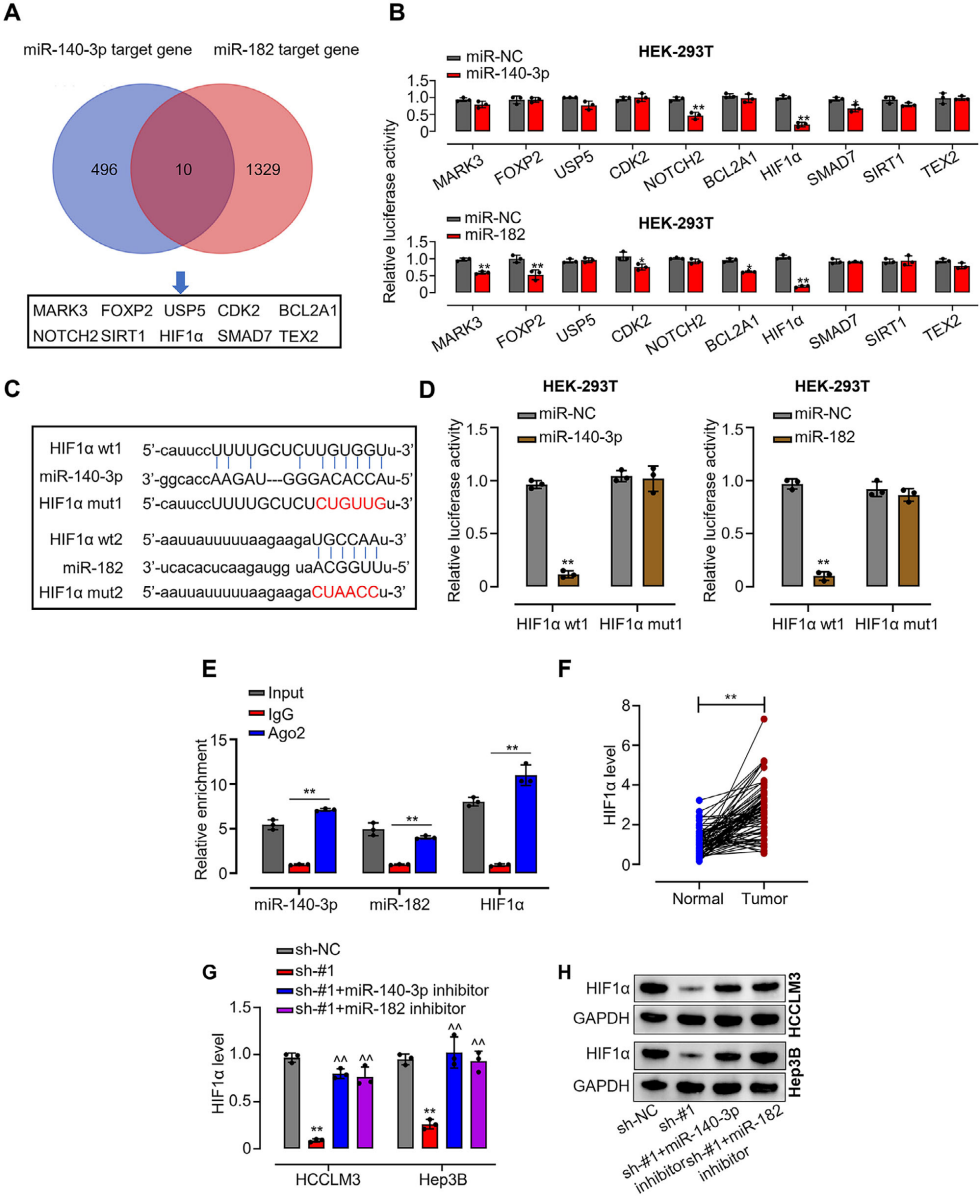
HIF1α is a direct target gene of miR‐140‐3p and miR‐182 in HCC. (A) A Venn diagram of miR‐140‐3p and miR‐182 target genes; (B) luciferase activity was determined in HEK293T cells after transfection with miR‐140‐3p/miR‐182 or miRNA negative control (miR‐NC) within the HIF1α promoter; (C) predicted binding sites of miR‐140‐3p and miR‐182 within HIF1α by bioinformatic analysis using the StarBase 3.0 (http://starbase.sysu.edu.cn/); (D) luciferase activity was determined in HEK293T cells after transfection with miR‐140‐3p/miR‐182 or miRNA negative control (miR‐NC); (E) RNA pull‐down assay was used to determine the interaction between HIF1α and miR‐140‐3p/miR‐182; (F) relative mRNA expression of HIF1α and miR‐140‐3p/miR‐182 in 80 pairs of HCC tissues and adjacent normal tissues was evaluated by qRT‐PCR; (G–I) mRNA and protein expression levels of HIF1α in HCCLM3 and Hep3B cells transfected with transfected with sh‐NC, sh‐circIST1#1, sh‐circIST1#1+ miR‐140‐3p, or miR‐182 inhibitor were evaluated using qRT‐PCR and western blotting assays. Data are representative of three independent experiments and shown as mean ± SD. *p < 0.05, **p < 0.01, compared to the miR‐NC or sh‐NC group; ^^*p* < 0.01, compared to the sh‐circIST1#1 group.

Bioinformatic screening (StarBase 3.0) for common targets of miR‐140‐3p and miR‐182 was performed to investigate the regulatory mechanism (Figure 5A). Functional validation using a luciferase reporter system identified HIF‐1α as the most strongly inhibited target, suggesting it is a primary functional endpoint of both miRNAs (Figure 5B). Bioinformatic analysis revealed binding sites in the HIF‐1α 3'UTR for both miR‐140‐3p and miR‐182 (Figure 5C). Subsequent luciferase assays confirmed that overexpression of miR‐140‐3p or miR‐182 significantly suppressed the activity of wild‐type HIF‐1α reporter, whereas this repression was attenuated upon mutation of the binding motifs in both HCCLM3 and Hep3B cells (Figure 5D). Furthermore, RNA pull‐down assays using a biotin‐labeled Ago2 probe demonstrated that miR‐140‐3p, miR‐182, and HIF‐1α mRNA were all enriched in Ago2‐containing ribonucleoprotein complexes (Figure 5E), supporting their functional association within the RISC complex. As shown in Figure 5F, qRT‐PCR analysis of 30 paired HCC clinical samples revealed that HIF‐1α was significantly upregulated in tumor tissues compared to adjacent non‐tumor tissues. Moreover, HIF‐1α expression showed a significant negative correlation with miR‐140‐3p and miR‐182 levels, and a positive correlation with circIST1 expression (Figure S1B). Consistently, knockdown of circIST1 markedly reduced HIF‐1α mRNA and protein levels in both HCCLM3 and Hep3B cells, as determined by qRT‐PCR and western blot analysis; however, this downregulation was effectively rescued upon co‐transfection with miR‐140‐3p or miR‐182 inhibitors (Figure 5G,H). Taken together, these results establish HIF‐1α as a direct downstream target of both miR‐140‐3p and miR‐182 in HCC.

### 2.6 circIST1 Promotes HCC Progression via HIF‐1α

**FIGURE 6:**
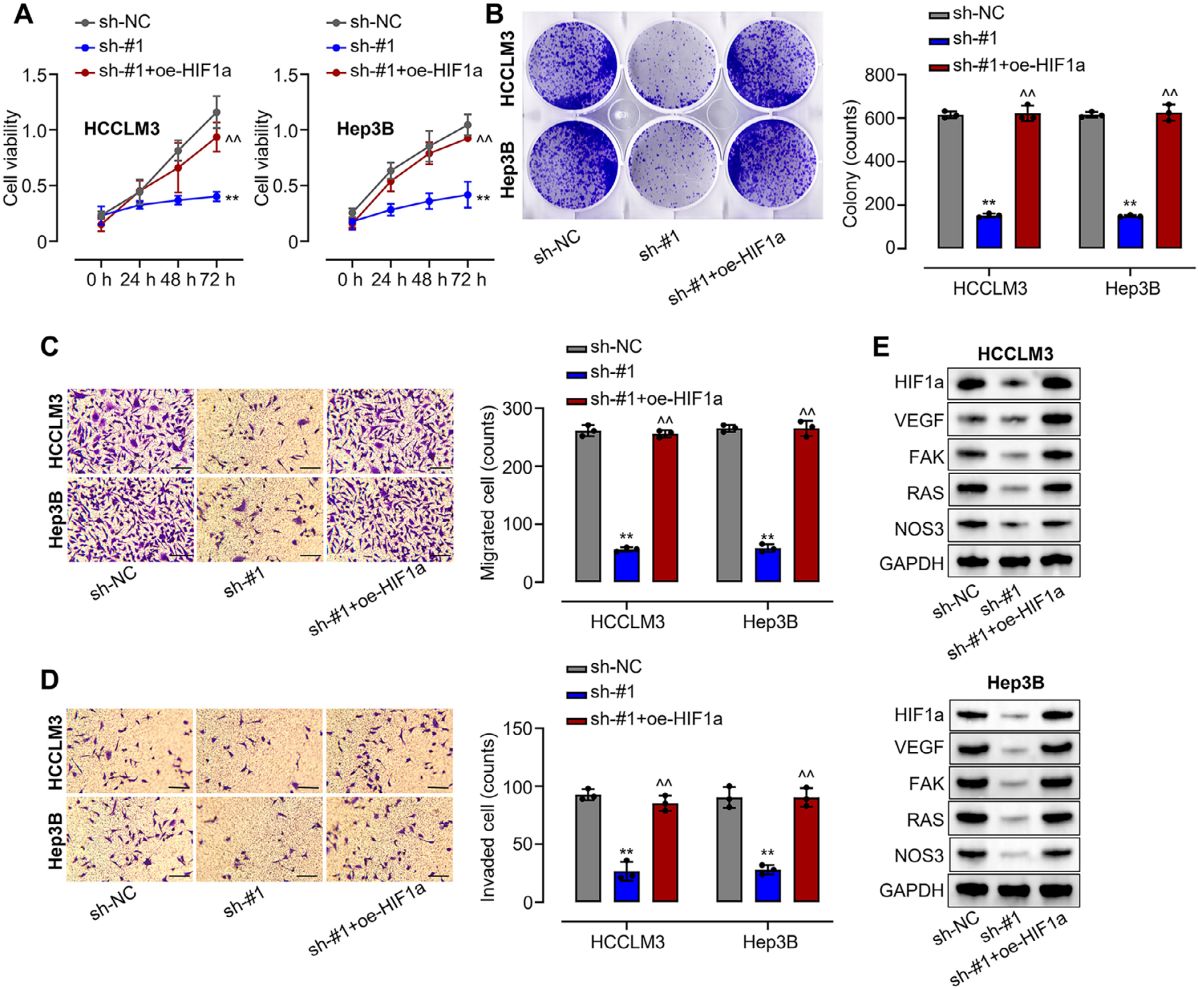
circIST1 depletion inhibits HCC progression via HIF1α. (A and B) CCK8 and colony formation assays was used to assess the proliferation of HCCLM3 and Hep3B cells transfected with sh‐NC, sh‐circIST1#1, sh‐circIST1#1+pcDNA3‐HIF1α; (C and D) Transwell assays were used to assess the migration and invasion of HCCLM3 and Hep3B cells transfected with sh‐NC, sh‐circIST1#1, sh‐circIST1#1+ pcDNA3‐HIF1α; (E) protein expression levels of HIF1α and its targets in HCCLM3 and Hep3B cells transfected with transfected with sh‐NC, sh‐circIST1#1, sh‐circIST1#1+ pcDNA3‐HIF1α were evaluated using western blotting assays. Data are representative of three independent experiments and shown as mean ± SD. **p < 0.01, compared to the sh‐NC group; ^^*p* < 0.01, compared to the sh‐circIST1#1 group.

To determine if circIST1 drives HCC progression through HIF‐1α, we restored HIF‐1α expression in circIST1‐knockdown HCCLM3 and Hep3B cells. HIF‐1α overexpression counteracted the suppression of proliferation caused by circIST1 silencing, as evidenced by CCK‐8 and colony formation assays (Figure 6A,B). It also reversed the reductions in cell migration and invasion observed upon circIST1 knockdown in both lines (Figure 6C,D). Furthermore, circIST1 knockdown decreased the protein levels of HIF‐1α and its downstream targets (Figure 6E). Together, these results demonstrate that circIST1 promotes HCC progression by activating the HIF‐1α signaling pathway.

### 2.7 circIST1 Promotes Tumor Glycolysis in HCC via Upregulation of HIF‐1α

**FIGURE 7:**
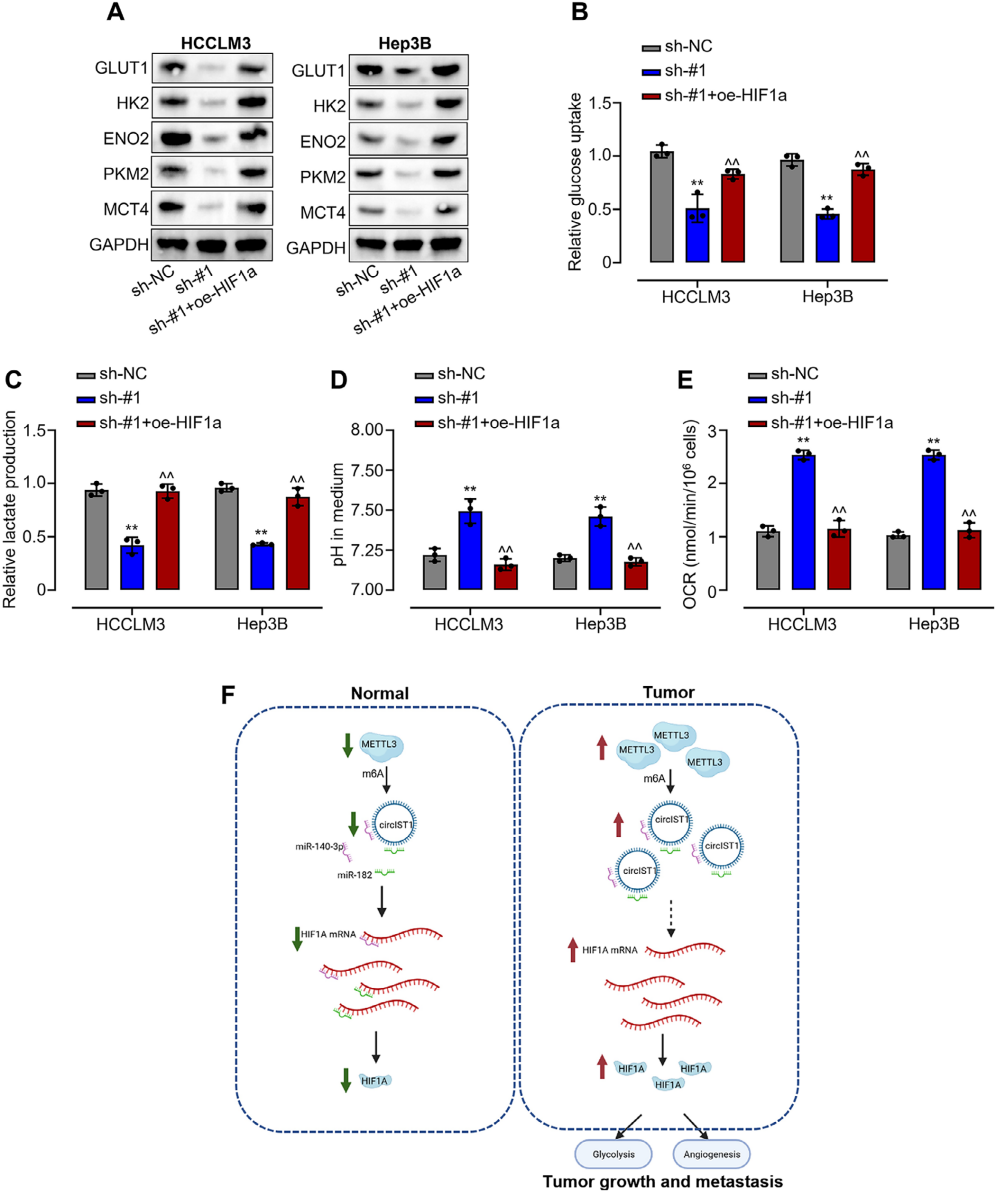
circIST1 downregulation disturbs tumor glycolysis in HCC via HIF1α. (A) Protein expression levels of glycolysis pathway targets in HCCLM3 and Hep3B cells transfected with transfected with sh‐NC, sh‐circIST1#1, sh‐circIST1#1+ pcDNA3‐HIF1α were evaluated using western blotting assays. (B) Glucose consumption, (C) lactate production, (D) pH in medium, in HCCLM3 and Hep3B cells transfected with transfected with sh‐NC, sh‐circIST1#1, sh‐circIST1#1+ pcDNA3‐HIF1α; (E) oxygen consumption rate (OCR) levels were measured by the Cell Mito Stress Test after cells transfected with sh‐NC, sh‐circIST1#1, sh‐circIST1#1+ pcDNA3‐HIF1α; (F) a schematic diagram that m6A modification of circIST1 promotes HCC progression by regulation of the miR‐140‐3p/miR‐182/HIF1α axis. Data are representative of three independent experiments and shown as mean ± SD. **p < 0.01, compared to the sh‐NC group; ^^*p* < 0.01, compared to the sh‐circIST1#1 group.

HIF‐1α, a key transcriptional regulator, controls the expression of numerous genes in glucose metabolism [31]. Given our findings that circIST1 promotes HCC progression via HIF‐1α, we further investigated the role of circIST1 in glycolysis. Western blot analysis showed that knockdown of circIST1 reduced the protein levels of key glycolytic enzymes downstream of HIF‐1α, and this reduction was partially rescued by HIF‐1α overexpression (Figure 7A). The functional consequences of circIST1 knockdown on cellular metabolism were assessed by measuring key glycolytic and mitochondrial parameters—including glucose uptake, lactate production, extracellular acidification rate (ECAR), and oxygen consumption rate (OCR)—in HCC cells. The results demonstrated that circIST1 silencing significantly impaired glycolytic metabolism, whereas HIF‐1α overexpression largely restored glycolytic function, as evidenced by recovery of glucose uptake, lactate production, medium pH (reflected by ECAR), and OCR levels (Figure 7B–E). These findings indicate that circIST1 regulates tumor glycolysis in HCC through the HIF‐1α signaling pathway.

## 3 Discussion

In this study, we identified a novel circRNA, circIST1, that is associated with HCC. Silencing circIST1 significantly suppresses HCC progression both in vitro and in vivo. Mechanistically, circIST1 functions as a ceRNA by endogenously sequestering two distinct miRNAs, miR‐140 and miR‐183‐3p that directly target HIF‐1α, thereby promoting HCC progression through upregulation of HIF‐1α (Figure 7F). These findings demonstrate that circIST1 exerts oncogenic effects during HCC development and highlights its potential as a therapeutic target via modulation of the HIF‐1α signaling pathway.

CircRNAs are cytoplasmic regulatory molecules that can serve as ceRNAs to modulate miRNA activity and depress target genes. Their dysregulation has been implicated in various stages of tumorigenesis, underscoring potential clinical utility as biomarkers or therapeutic targets [32, 33]. Notably, many circRNAs display expression patterns that are highly specific to certain tissues or cancer types [34, 35]. Notably, a single circRNA can exert distinct functional mechanisms within the same tumor type. For example, ciRS‐7 promotes lung cancer progression through the miR‐7/RELA signaling pathway [36], while other studies have shown that ciRS‐7 also drives carcinogenesis via the miR‐219a/SOX5 axis in the same cancer type [37]. Moreover, the functional roles of a given circRNA can vary across different tumor types. In osteosarcoma (OS), ciR‐ITCH exerts oncogenic effects by activating the miR‐7/EGFR pathway, whereas in bladder cancer, it acts as a tumor suppressor through the miR‐17/miR‐224/p21/PTEN axis [38, 39]. In this study, we identify circIST1 as a novel, highly expressed circRNA in HCC. It functions as a ceRNA by sequestering miR‐140 and miR‐180‐3p, thereby promoting HCC progression. Given these context‐dependent roles of circRNAs, further investigation is warranted to determine whether circIST1 exhibits divergent regulatory functions in different cancer types. This is particularly important in HCC, where circIST1 may influence tumor progression through multiple mechanisms. Indeed, circRNAs are known to perform diverse biological functions beyond their role as miRNA sponges, including interactions with RNA‐binding proteins, modulation of transcription, and even serving as templates for peptide translation [40, 41, 42]. Elucidating the multifaceted roles of circIST1 may provide deeper insights into its functional plasticity and therapeutic potential.

Dysregulated expression of circRNA is frequently driven by epigenetic mechanisms, including DNA methylation of parental genes, histone modifications, and RNA methylation [43, 44]. Among these, m6A RNA methylation represents the most prevalent mechanism regulating circRNA biogenesis and expression. Our previous study also identified a specific circRNA whose expression was upregulated through m6A methylation [18]. m6A RNA methylation is dynamically regulated by three functionally distinct classes of proteins. First, RNA methyltransferases known as “Writers”, including METTL3, METTL14, and WTAP, catalyze the deposition of m6A modifications. Second, m6A demethylases termed “Erasers”, such as FTO and ALKBH5, mediate the removal of m6A marks. Finally, m6A‐binding proteins referred to as “Readers”, including members of the YTH domain family, IGF2BP family, and hnRNPs, recognize and bind to m6A‐modified sites, thereby translating the methylation signal into functional outcomes [45]. Accumulating evidence indicates that m6A methylation generally enhances the stability of circRNAs, contributing to their regulatory roles in cellular processes [46, 47]. Our data indicate that circIST1 undergoes significant m6A methylation modifications mediated by METTL3. This modification markedly enhances the stability and prolongs the cytoplasmic half‐life of circIST1, leading to its upregulated expression. Upon recognition of m6A‐modified RNAs by reader proteins, two opposing fates can ensue: either increased translational efficiency and RNA stability or targeted degradation [48]. In this study, although we did not specifically identify the m6A reader proteins responsible for circIST1 regulation, our findings suggest that m6A modification stabilizes circIST1. Notably, this effect may be mediated by the IGF2BP family, which has been previously shown to stabilize m6A‐modified RNAs. Nevertheless, further investigation is required to validate this hypothesis.

The heterodimeric transcription factor HIF‐1, composed of HIF‐1α and HIF‐1β subunits, orchestrates the transcriptional activation of numerous genes implicated in tumor angiogenesis, glucose metabolism, cellular survival, and invasion processes [13]. Consequently, HIF‐1α functions as a canonical oncogene that critically promotes malignant progression. Elevated HIF‐1α expression is consistently observed across diverse tumor types, with HCC representing a prominent example of this dysregulation [49]. This transcriptional upregulation primarily stems from the hypoxic TME and genetic alterations in key regulatory genes, including ERBB2, VHL, and PTEN [13]. In HCC, HIF‐1α functions as a central mediator within hypoxic niches, where it initiates tumor autophagy and augments cancer stemness properties [50, 51]. Furthermore, HIF‐1α masterfully coordinates the metabolic reprogramming of glucose utilization pathways in HCC, thereby accelerating the malignant progression of HCC [51]. Beyond transcriptional control mechanisms, non‐coding RNAs, particularly miRNAs, exert crucial regulatory influence on HIF‐1α at the post‐transcriptional level. Substantial evidence demonstrates that multiple miRNAs, including miR‐20b, miR‐21, miR‐451, miR‐671‐5p, and miR‐200c, directly target HIF‐1α transcripts, thereby constraining its post‐transcriptional translation [52, 53, 54, 55, 56, 57]. Consequently, miRNAs‐based approaches represent promising therapeutic strategies for modulating HIF‐1α expression. In the present study, we have identified two additional miRNAs, miR‐140 and miR‐180‐3p, that coordinately target HIF‐1α in HCC, expanding the regulatory network governing expression in this malignancy. HIF‐1α, functioning as a transcription factor that governs GLUT1 expression, enhances glucose uptake in tumor cells and promotes aerobic glycolysis when overexpressed, a mechanistic insight corroborated by our previous investigations [13, 40]. Suppression of glycolytic processes not only constrains tumor proliferation but also remodels the TME and attenuates immune evasion mechanisms [18]. Given the fundamental role of HIF‐1α in tumorigenesis, small molecule inhibitors targeting this transcription factor have been developed and are currently employed in clinical oncology practice [58]. Nevertheless, achieving optimal therapeutic efficacy remains a substantial clinical challenge. In the present study, we proposed an innovative therapeutic paradigm that modulates HIF‐1α expression through circIST1‐mediated sequestration of two distinct miRNAs that target HIF‐1α. Genetic ablation of circIST1 substantially attenuates HIF‐1α expression. Relative to conventional small‐molecule inhibitors, this RNA‐based intervention may provide a more nuanced and dependable therapeutic alternative. Notwithstanding the innovative mechanistic insights elucidated in our investigation, several methodological limitations merit explicit acknowledgment. Primarily, the clinical translational potential of the m6A‐circIST1‐miR‐140‐3p/miR‐182‐HIF‐1α regulatory axis necessitates comprehensive validation across expanded, multi‐institutional patient cohorts encompassing diverse etiological backgrounds and disease staging. Although our current sample size demonstrates adequacy for initial discovery‐phase analyses, it may constrain statistical robustness for detailed subgroup stratifications. Second, while we have established that METTL3‐catalyzed m6A modification augments circIST1 stability, the specific m6A “reader” proteins mediating recognition of m6A‐modified circIST1 remain molecularly uncharacterized. Subsequent investigations incorporating RNA pull‐down methodologies integrated with mass spectrometry proteomic analyses will be imperative to delineate these critical interacting partners.

In summary, our investigation has identified a previously uncharacterized HCC‐associated circRNA, designated circIST1, and delineated its mechanistic role in augmenting HIF‐1α expression through disruption of post‐transcriptional regulatory constraints. This circIST1‐mediated HIF‐1α upregulation subsequently orchestrates HIF‐1α‐driven aerobic glycolysis, thereby substantively contributing to HCC progression.

## 4 Materials and Methods

### 4.1 Clinical Patient Samples

A total of 80 paired HCC and adjacent non‐tumorous liver tissues were collected from treatment‐naïve patients diagnosed with primary HCC and undergoing radical hepatectomy at the Third Affiliated Hospital of Navy Military Medical University. All tissues were snap‐frozen within 30 min post‐resection and maintained at −80°C until molecular analysis.

### 4.2 Cell Culture and Transfection

A normal human hepatic epithelial cell line (L02) and four HCC cell lines, including Huh7, HepG2, HCCLM3, and Hep3B, were obtained from the Cell Bank of the Type Culture Collection, Chinese Academy of Sciences (Shanghai, China). Cells were cultured in Dulbecco's Modified Eagle's Medium (DMEM; Gibco, USA) supplemented with 10% (v/v) fetal bovine serum (FBS) and maintained in a humidified incubator at 37°C with a 5% CO_2_. To overexpress circIST1 or HIF‐1α, the full‐length sequences of circIST1 or HIF‐1α were cloned into the pcDNA3.1 vector (Invitrogen, CA, USA), generating the recombinant plasmids pcDNA3.1‐circIST1 and pcDNA3.1‐HIF‐1α. Plasmid construction was performed according to previously published protocols from our group [40]. For circIST1 knockdown, small interfering RNAs (siRNAs) targeting circIST1 or non‐targeting negative control (NC) sequences were cloned into the pLKO.1 plasmid (YouBio, China), followed by transfection into HCCLM3 and Hep3B cells. Stable knockdown cell lines were generated via puromycin selection. All miRNA mimics (miR‐140‐3p and miR‐182), inhibitors, and corresponding NCs were procured from GeneCopoeia (Guangdong, China). Following the manufacturer's protocol, transfections were carried out with Lipofectamine 2000 (Invitrogen, USA), and cells were collected 24 h later for subsequent analysis.

### 4.3 Cell Treatment

In the control group, HCC cells were exposed to DMSO or to a panel of compounds (Actinomycin D, 5‐aza‐2’‐deoxycytidine, PCI‐24781, RGFP966, ACY‐1215, and SAHA) at 5 µM. All compounds were sourced from Sigma (USA). After a 6‐h incubation, cells were processed for RNA extraction and subsequent circIST1 evaluation.

### 4.4 qRT‐PCR

Total RNA was isolated from HCC cell lines with TRIzol reagent (Invitrogen, USA) following the manufacturer's protocol. Following extraction, RNA was reverse‐transcribed into cDNA using a High‐Capacity cDNA Reverse Transcription Kit (Applied Biosystems, USA). Subsequent qRT‐PCR was conducted on an ABI 7900HT system (Applied Biosystems, USA) using Power SYBR Green PCR Master Mix and gene‐specific primers. mRNA and miRNA levels were normalized to GAPDH and U6 snRNA, respectively, and relative expression was quantified via the 2^−ΔΔCt^ method.

### 4.5 RNase R Treatment

Linear RNA depletion was performed by incubating 2 µg total RNA with RNase R (Epicentre, USA) in a standard 20 µL reaction buffer at 37°C for 45 min; control reactions used nuclease‐free water instead of the enzyme. After heat inactivation (70°C, 10 min), the RNA was directly subjected to qRT‐PCR analysis.

### 4.6 Gene‐Specific m6A qRT‐PCR

m6A modifications in *circIST1* were analyzed using the riboMeRIP m6A Transcriptome Profiling Kit (Ribobio, China) according to the manufacturer's instructions. Total RNA was extracted using TRIzol reagent (Invitrogen, USA), and 50–100 µg of RNA was fragmented into ∼100‐nucleotide fragments by chemical or thermal fragmentation. Of the fragmented RNA, 90% was subjected to immunoprecipitation by overnight incubation at 4°C with protein A/G beads conjugated to either an anti‐m6A antibody or mouse IgG (NC), while 10% was reserved as input control. Following incubation, m6A‐containing RNA was eluted from the anti‐m6A immunoprecipitate (or IgG control) through competitive displacement with free m6A nucleoside. The enriched RNA fractions and input samples were then analyzed by qRT‐PCR to assess the m6A enrichment of circIST1 relative to the input.

### 4.7 Bioinformatic Analysis

To identify HCC‐associated circRNAs, we retrieved circRNA expression profiles from the Gene Expression Omnibus (GEO) database (Accession: GSE164803; https://www.ncbi.nlm.nih.gov/geo/query/acc.cgi?acc = GSE164803). This dataset includes expression levels of 3045 unique circRNAs derived from six paired HCC tissues and their matched adjacent non‐tumorous liver tissues. Differentially expressed circRNAs were identified using the limma package in R, with thresholds of |log_2_ fold change| > 1 and adjusted *p*‐value < 0.05 considered statistically significant. The top 20 most significantly upregulated and downregulated circRNAs were visualized using the Heatmap package in R. All differentially expressed circRNAs were further depicted using the ggplot2 package for comprehensive profiling. The potential binding sites of miR‐140 and miR‐182 mimics within circIST1 and HIF‐1α were predicted using StarBase 3.0 (http://starbase.sysu.edu.cn/) following standard protocols.

### 4.8 CCK‐8 Assay

HCC cells, transfected and treated according to the specified protocols, were seeded into 96‐well plates at a density of 3000 cells per well. Cells were incubated for 0, 24, 48, and 72 h under standard culture conditions. At each time point, 10 µL of CCK‐8 solution (Dojindo, Japan) was added to each well, followed by incubation at 37°C for 1 h. The absorbance was then measured at 450 nm using a microplate reader (Synergy H4 Hybrid Reader, BioTek, USA).

### 4.9 Colony Formation Assay

HCCLM3 or Hep3B cells (500 cells per well) were seeded into 6‐well plates and transfected or treated as specified. After approximately 10 days of culture, cells were fixed with methanol and stained with crystal violet (Beyotime, China) for 30 min. Colonies were then imaged using a Nikon Inverted Research Microscope Eclipse Ti, and colony numbers were quantified using ImageJ software. Raw data from three independent experiments are presented in Figure S2.

### 4.10 Transwell Assay

To evaluate cellular migration and invasion, Transwell assays were performed. After transfection and treatment, 1 × 10^5^ cells in 500 µL of serum‐free medium were seeded into the upper chamber. For invasion assays, the membrane was pre‐coated with Matrigel (BD, USA); for migration assays, uncoated membranes were used. The lower chamber was filled with 700 µL of complete medium supplemented with 10% FBS and placed in a 12‐well plate. After 24 h at 37°C, cells on the upper membrane surface were removed with cotton swabs. Cells that had traversed the membrane were then fixed with methanol, stained with crystal violet (Beyotime, China), and imaged using a Nikon Eclipse Ti microscope. Cell counts from six random fields per well were quantified. Data from three independent experiments are shown in Figure S3.

### 4.11 Apoptosis Assay

HCCLM3 or Hep3B cells were transfected and seeded into six‐well plates at a density of 1 × 10^6^ cells per well, followed by designated treatments. After treatment, cells were harvested by centrifugation at 1500 × *g* for 5 min, washed once with cold PBS, and then resuspended in 1× binding buffer. Cells were incubated with 5 µL of FITC‐conjugated Annexin V and 5 µL of propidium iodide (PI) for 30 min in the dark at 4°C. Flow cytometric analysis was performed using a BD FACS Aria II instrument (BD Biosciences, USA). Gating strategies and compensation controls were applied as described in the previous study [18].

### 4.12 Western Blot Analysis

Following treatment, HCCLM3 and Hep3B cells were lysed in RIPA buffer, and the total protein concentration was quantified using a BCA Protein Assay Kit (Beyotime, China). Equal amounts of protein were separated by SDS‐PAGE, transferred to PVDF membranes (Millipore, USA), and then blocked with 5% non‐fat milk in TBST for 1 h at room temperature. Subsequently, membranes were incubated overnight at 4°C with specific primary antibodies from Abcam (UK) against METTL3 (ab195352), HIF‐1α (ab308433), VEGF (ab32152), FAK (ab40794), RAS (ab108602), NOS3 (ab300071), GLUT1 (ab115730), HK2 (ab209847), ENO2 (ab222514), PKM2 (ab85555), and MCT4 (ab308528), using GAPDH (ab181602) as the loading control. After three washes with TBST, membranes were incubated with HRP‐conjugated secondary antibodies. Protein bands were finally visualized using Immobilon Western Chemiluminescent HRP Substrate (Millipore, USA) and detected via chemiluminescence imaging.

### 4.13 Luciferase Reporter Assays

On the day prior to transfection, HCCLM3 or Hep3B cells were seeded into 24‐well plates at a density of 2 × 10^4^ cells per well. Cells were then transfected with either wild‐type or mutant reporter plasmids using Lipofectamine 2000 (Invitrogen, USA) according to the manufacturer's instructions. Twenty‐four hours post‐transfection, luciferase activity was measured using the Dual‐Luciferase Reporter Assay System (Promega, USA). Firefly luciferase signals were normalized to Revilla luciferase activity for relative reporter activity.

### 4.14 In Vivo Xenograft Experiments

Mice (male, BALB/c nude, 6 weeks old; *n* = 6 per group) were used to establish subcutaneous xenografts. In compliance with ethical standards, all animal procedures were performed according to protocols approved by the IACUC of Shanghai Eastern Hepatobiliary Surgery Hospital. A total of 1 × 10^6^ circIST1‐knockdown or NC Hep3B cells were suspended in PBS or Matrigel and injected subcutaneously into the right flank of each mouse. Mice were monitored daily for general health and tumor formation. Tumor growth was measured with calipers starting on day 7 post‐inoculation and every 7 days thereafter. Tumor volume was calculated using the formula: Tumor volume (mm^3^) = length × (width)^2^/2, where length represents the longest dimension and width the shortest perpendicular dimension. On day 35, mice were euthanized, and tumors were surgically excised, weighed, and recorded. Tumor tissues were immediately snap‐frozen in liquid nitrogen and stored at −80°C for subsequent molecular analyses.

### 4.15 RNA Pull‐Down‐qRT‐PCR Assays

For RNA pull‐down assays, custom biotin‐labeled probes (circIST1‐specific and NC) were obtained from GenePharma (Shanghai, China). After transfection into HCCLM3 and Hep3B cells, the probes were incubated with cells at 4°C for 48 h to allow for binding under controlled, non‐physiological conditions. Following transfection, cells were harvested, lysed, and incubated with streptavidin‐conjugated magnetic beads (Invitrogen, USA) at room temperature (25°C) for 2 h to capture biotin‐bound RNA complexes. After stringent washing and elution using an appropriate binding buffer, the RNAs specifically associated with the probes were isolated and subjected to qRT‐PCR analysis for quantification.

### 4.16 Glucose Uptake

Following cell transfection and treatment, glucose uptake was assessed using the glucose oxidase‐peroxidase assay coupled with Amplex Red reagent oxidation (Sigma, USA), according to the manufacturer's protocol. Briefly, the reaction was terminated by washing cells three times with ice‐cold PBS. Cells were then lysed in lysis buffer, and intracellular glucose levels were quantified using a Glucose Uptake Assay Kit (K682‐50, BioVision, CA, USA) following the manufacturer's instructions. Fluorescence or absorbance was measured at an excitation/emission wavelength of 544/590 nm or at 412 nm, respectively, using a microplate reader. A standard curve was generated from DMEM supplemented with known concentrations of glucose to calibrate glucose content. To determine cellular glucose consumption, the glucose concentration in cell‐free culture medium (background control) was subtracted from that in conditioned medium. The relative glucose uptake rate was calculated based on the change in glucose mass (or molar amount) over time and normalized to the control group.

### 4.17 Oxygen Consumption Rate Detection

OCR was assessed in real time on a Seahorse XF96 Extracellular Flux Analyzer (Seahorse Bioscience, Billerica, USA) as per the standard protocol. Cells were seeded in XF96 microplates and incubated in assay medium (unbuffered DMEM, pH 7.4) at 37°C in a non‐CO_2_ incubator. OCR was assessed under basal conditions and following sequential injections of oligomycin, FCCP, and rotenone/antimycin A. Data are expressed as pmol O_2_/min per well and normalized to cell number or protein content.

### 4.18 Lactic Acid Detection

Lactate levels in cell culture supernatants were quantified using the CheKine Micro Lactate Assay Kit (Abbkine, USA). Briefly, following 24‐h culture after circIST1 knockdown and HIF‐1α overexpression (or NC), supernatants from HCCLM3 and Hep3B cells were collected. Lactate concentration was determined spectrophotometrically based on the lactate oxidase‐mediated colorimetric reaction, with absorbance measured at 570 nm. Concentrations were interpolated from a standard curve and expressed as µmol/10⁶ cells or µmol/mg protein, normalized to the control group.

### 4.19 Cell Supernatant PH Detection

The pH of cell culture supernatants was measured using a benchtop pH meter (METTLER TOLEDO) to assess extracellular acidity. HCCLM3 or Hep3B cells were cultured for 24 h following circIST1 knockdown, HIF‐1α overexpression, or NC treatment. Supernatants were collected and analyzed immediately using a calibrated pH electrode. The instrument was calibrated with a standard pH 7.0 buffer solution prior to measurement, and readings were obtained according to the manufacturer's operational guidelines. All measurements were performed at room temperature with gentle stirring to ensure accuracy and reproducibility.

### 4.20 Statistical Analysis

We performed statistical analysis with GraphPad Prism 8 (GraphPad Software, Inc., USA). An unpaired two‐tailed Student's *t*‐test was used for two‐group comparisons, while one‐way ANOVA followed by Tukey's post hoc test was applied for multi‐group analyses. Results from three independent biological replicates are presented as mean ± SD. A *p*‐value below 0.05 was considered significant.

## Author Contributions

Study concept and design: Y. Zhan, Z. Wang, F. Teng. Data collection: Q. Ding, L. Lv, F. Xie, and Y. Huang. Analysis and interpretation of results: X. Jiang, D. Zheng, and X. Ge. Draft manuscript preparation: S. Cheng, Y. Zhu, and L. Bao. All authors have read and approved the final manuscript.

## Funding

This study was supported by the Shanghai Natural Science Foundation (23ZR1478100 to Leilei Bao and 25ZR1402579 to Yangyang Zhan), “Soaring Project” Talent Development Program of Eastern Hepatobiliary Surgery Hospital (TF2024LYLY03 to Leilei Bao and TF2024XSYJ08 to Yangyang Zhan), Naval Medical University University‐level Scientific Research Startup Fund (2023MS040 to Leile Bao and 2023QN092 to Yangyang Zhan), Youth Cultivation Fund of Eastern Hepatobiliary Surgery Hospital (2022GZR001 to Yangyang Zhan).

## Ethics Statement

This study was approved by the Ethics Committee of the Shanghai Eastern Hepatobiliary Surgery Hospital (project number EHBHKY2022‐K003‐P001).

## Conflicts of Interest

Yizhun Zhu is an editorial board member of *MedComm*. Yizhun Zhu was not involved in the journal's review of or decisions related to this manuscript. The other authors declare no conflicts of interest.

## Supporting information




**Figure S1**. (A) qRT‐PCR assay was used to detect the expression of miR‐140‐3p/miR‐180 in HCCLM3 and Hep 3B transfected with sh‐NC, sh‐circIST1#1, sh‐circIST1#1+ miR‐140‐3p or miR‐182 inhibitor. (B) Pearson correlation analysis was performed to analyze the expression correlation between miR‐140‐3p/miR‐180, circIST1 and HIF‐1α. (C) The tumor images, and their growth curves of nude mice with sh‐NC or sh‐circIST1#1 Hep3B cells; tumor weights of each group were analyzed at the endpoint of the experiment. Data are representative of three independent experiments and shown as mean ± SD. **p<0.01, compared to sh‐#1.
**Figure S2**. The original data of three independent experiment repeats of colony formations.
**Figure S3**. The original data of three independent experiment repeats of Transwells.
**Figure S4**. Predicted binding sites of miR‐140‐3p and miR‐182 within HIF1α by bioinformatic analysis using the StarBase 3.0 (Left panel). Luciferase activity was determined in HEK293T cells after transfection with miR‐140‐3p/miR‐182, mutant miR‐140‐3p/miR‐182 or miRNA negative control (miR‐NC). **p<0.01.
**Table S1**. Association between clinical features and circIST1 expression of hepatocellular carcinoma patients.

## Data Availability

All data used and analyzed in this study are available from the corresponding author upon reasonable request. Email should be sent to annabao212@126.com to obtain the shared data.
