# Supplementary material for: N6‐Methyladenosine Modification of circIST1 Promotes Hypoxia‐Inducible Factor α–mediated Glycolysis and Progression in Hepatocellular Carcinoma
Source: MedComm (2020). 2026 Jan 14;7(1):e70577. doi: 10.1002/mco2.70577 (PMC12801398; doi:10.1002/mco2.70577)
Supplement: Supplementary file 1 — Figure S1. (A) qRT‐PCR assay was used to detect the expression of miR‐140‐3p/miR‐180 in HCCLM3 and Hep 3B transfected with sh‐NC, sh‐circIST1#1, sh‐circIST1#1+ miR‐140‐3p or miR‐182 inhibitor. (B) Pearson correlation analysis was performed to analyze the expression correlation between miR‐140‐3p/miR‐180, circIST1 and HIF‐1α. (C) The tumor images, and their growth curves of nude mice with sh‐NC or sh‐circIST1#1 Hep3B cells; tumor weights of each group were analyzed at the endpoint of the experiment. Data are representative of three independent experiments and shown as mean ± SD. **p<0.01, compared to sh‐#1. Figure S2. The original data of three independent experiment repeats of colony formations. Figure S3. The original data of three independent experiment repeats of Transwells. Figure S4. Predicted binding sites of miR‐140‐3p and miR‐182 within HIF1α by bioinformatic analysis using the StarBase 3.0 (Left panel). Luciferase activity was determined in HEK293T cells after transfection with miR‐140‐3p/miR‐182, mutant miR‐140‐3p/miR‐182 or miRNA negative control (miR‐NC). **p<0.01. Table S1. Association between clinical features and circIST1 expression of hepatocellular carcinoma patients. [file MCO2-7-e70577-s001.docx]

**Title:** **N6-methyladenosine modification of novol circIST1 promotes HIF-1**α **mediated hepatocellular carcinoma glycolysis and cancerous progression**

Yangyang Zhan^1, *^, Zhongmin Wang^1, *^, Fei Teng^3, *^, Qian Ding^2^, Lei Lv^1^, Fangyuan Xie^1^, Yueying Huang^1^, Xue Jiang^1^, Dan Zheng^1^, Xiaoying Ge^1^, Shuqun Cheng^4, #^, Yizhun Zhu^2,#^, Leilei Bao^1,#^.

^1^Department of Pharmacy, Shanghai Eastern Hepatobiliary Surgery Hospital, Navy Military Medical University, 225 Changhai Road, Yangpu District, Shanghai, China.

^2^State Key Laboratory of Quality Research in Chinese Medicine, School of Pharmacy, Macau University of Science and Technology, Avenida Wai Long, Taipa, Macau, China.

^3^Department of Liver Surgery and Organ Transplantation, Changzheng Hospital, Naval Medical University, 415 Fengyang Road, Huangpu District, Shanghai, China.

^4^Department of Hepatic Surgery VI, Eastern Hepatobiliary Surgery Hospital, Navy Military Medical University, 225 Changhai Road, Yangpu District, Shanghai, China.

^*^These authors contributed equally to this works.

^#^ Correspondence: annabao212@126.com (Leilei Bao), yzzhu@must.edu.mo (Yizhun Zhu), [chengshuqun@aliyun.com](mailto:chengshuqun@aliyun.com) (Shuqun Cheng).


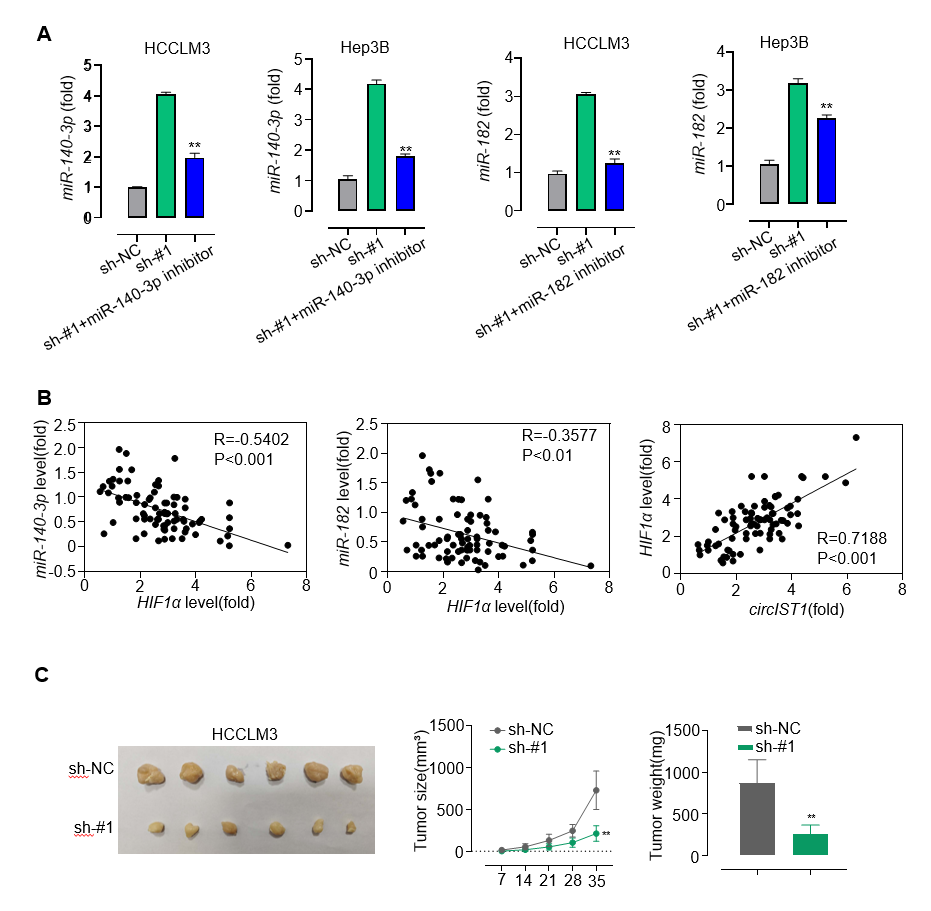


**Figure S1.** (A) qRT-PCR assay was used to detect the expression of miR-140-3p/miR-180 in HCCLM3 and Hep 3B transfected with sh-NC, sh-circIST1#1, sh-circIST1#1+ miR-140-3p or miR-182 inhibitor. (B) Pearson correlation analysis was performed to analyze the expression correlation between miR-140-3p/miR-180, circIST1 and HIF-1α. (C) The tumor images, and their growth curves of nude mice with sh-NC or sh-circIST1#1 Hep3B cells; tumor weights of each group were analyzed at the endpoint of the experiment. Data are representative of three independent experiments and shown as mean ± SD. **p＜0.01, compared to sh-#1.


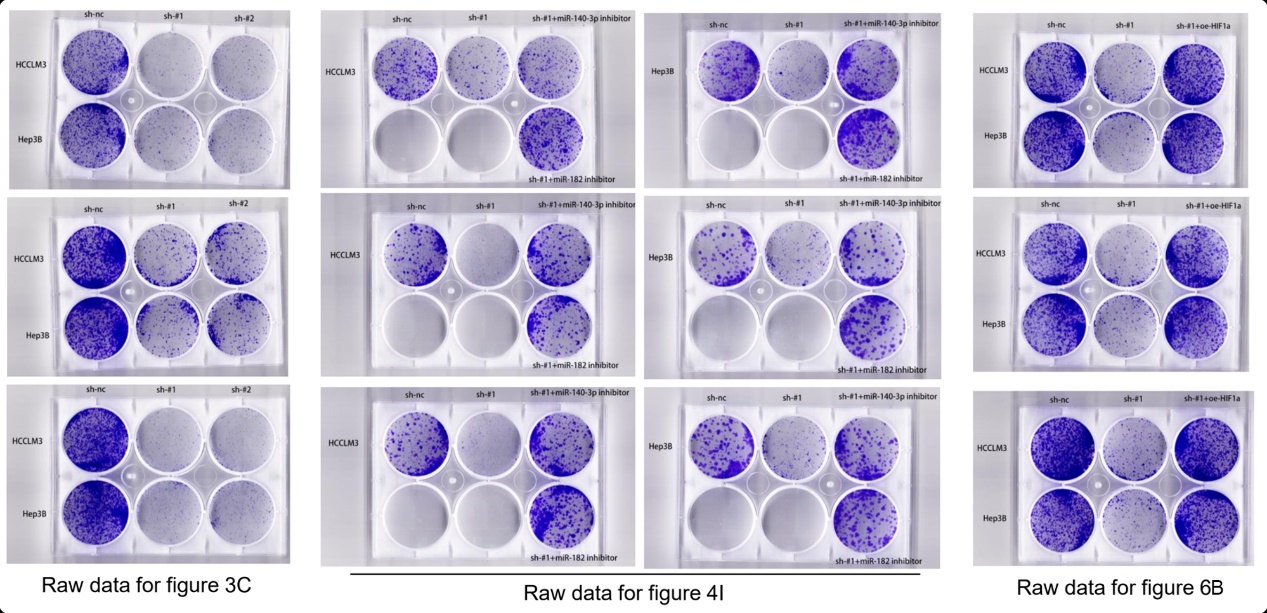


**Figure S2**

**The original data of three independent experiment repeats of colony formations.**


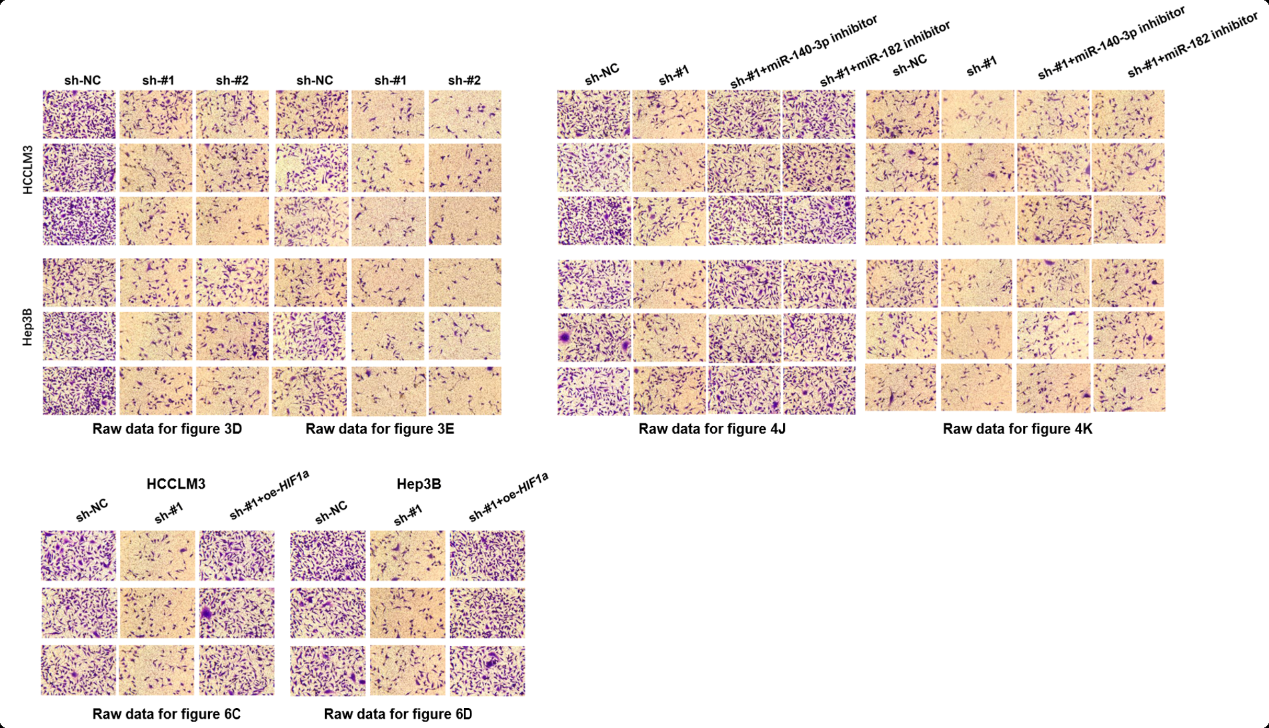


**Figure S3**

**The original data of three independent experiment repeats of Transwells.**

**
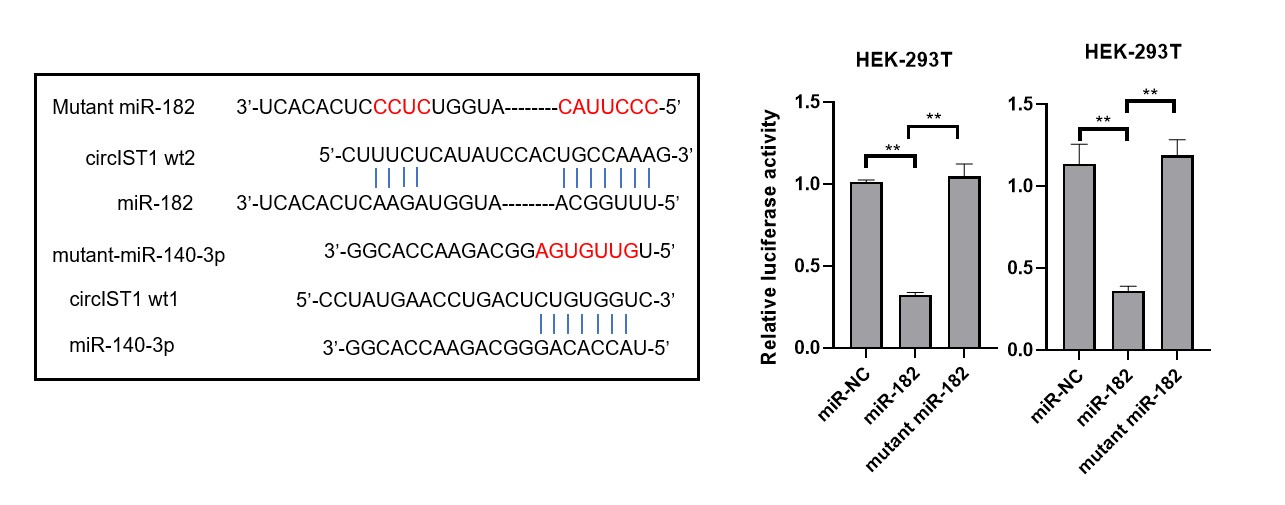
**

**Figure S4.** Predicted binding sites of miR-140-3p and miR-182 within HIF1α by bioinformatic analysis using the StarBase 3.0 (Left panel). Luciferase activity was determined in HEK293T cells after transfection with miR-140-3p/miR-182, mutant miR-140-3p/miR-182 or miRNA negative control (miR-NC). **p＜0.01.

**Table S1.** Association between clinical features and circIST1 expression of hepatocellular carcinoma patients.

| Chinicopathological  characteristics | Total  (80) | CircIST1  Low expression  (40) | CircIST1  High expression  (40) | X^2^ | P value |
| --- | --- | --- | --- | --- | --- |
| Gender |  |  |  |  |  |
| male | 51 | 27 | 24 | 0.16 | 0.583 |
| female | 29 | 13 | 16 |  |  |
| Age |  |  |  |  |  |
| <=50 | 43 | 19 | 24 | 1.257 | 0.185 |
| >50 | 37 | 21 | 16 |  |  |
| Tumor size |  |  |  |  |  |
| Large（≥30mm） | 31 | 16 | 25 | 4.053 | 0.044* |
| Small（＜30mm） | 49 | 24 | 15 |  |  |
| Tumor number |  |  |  |  |  |
| ＞1 | 20 | 8 | 12 | 1.067 | 0.302 |
| 1 | 60 | 32 | 28 |  |  |
| HBV |  |  |  |  |  |
| + | 31 | 12 | 19 | 2.581 | 0.108 |
| - | 49 | 28 | 21 |  |  |
| Fibrosis |  |  |  |  |  |
| + | 33 | 15 | 18 | 0.464 | 0.496 |
| - | 47 | 25 | 22 |  |  |
| Differentiation |  |  |  |  |  |
| high | 29 | 15 | 14 | 1.641 | 0.440 |
| moderate | 20 | 12 | 8 |  |  |
| poor | 31 | 13 | 18 |  |  |
| Lymph node metastasis |  |  |  |  |  |
| Positive | 38 | 13 | 25 | 7.218 | 0.007 |
| Negative | 42 | 27 | 15 |  |  |
| TMN stages |  |  |  |  |  |
| I+ II | 44 | 26 | 18 | 3.905 | 0.048* |
| III+IV | 36 | 14 | 22 |  |  |
| intrahepatic metastasis |  |  |  |  |  |
| Positive | 39 | 14 | 25 | 6.054 | 0.014* |
| Negative | 41 | 26 | 15 |  |  |
| distant metastasis |  |  |  |  |  |
| Positive | 45 | 16 | 29 | 8.584 | 0.003** |
| Negative | 35 | 24 | 11 |  |  |
